# Maternal High Fat Diet Consumption Exaggerates Metabolic Disorders in Mice With Cigarette-Smoking Induced Intrauterine Undernutrition

**DOI:** 10.3389/fnut.2021.638576

**Published:** 2021-03-16

**Authors:** Taida Huang, Mo Yang, Yunxin Zeng, Xiaomin Huang, Nan Wang, Yun Chen, Peng Li, Jinqiu Yuan, Chun Chen, Brian G. Oliver, Chenju Yi

**Affiliations:** ^1^Research Center, The Seventh Affiliated Hospital of Sun Yat-sen University, Shenzhen, China; ^2^Faculty of Medicine, School of Biomedical Sciences, The Chinese University of Hong Kong, Hong Kong, China; ^3^Faculty of Science, School of Life Sciences, University of Technology Sydney, Sydney, NSW, Australia; ^4^Respiratory Cellular and Molecular Biology, Woolcock Institute of Medical Research, Sydney, NSW, Australia

**Keywords:** smoke exposure, high fat diet, feeding regulation, glucose intolerance, myogenes

## Abstract

**Objectives:** Maternal smoking causes fetal underdevelopment and results in births which are small for gestation age due to intrauterine undernutrition, leading to various metabolic disorders in adulthood. Furthermore, postnatal high fat diet (HFD) consumption is also a potent obesogenic factor, which can interact with maternal smoking. In this study, we aimed to determine whether maternal HFD consumption during pregnancy can reverse the adverse impact of maternal smoking and change the response to postnatal HFD consumption.

**Methods:** Female mice were exposed to cigarette smoke (SE, 2 cigarettes/day) or sham exposed for 5 weeks before mating, with half of the SE dams fed HFD (43% fat, SE+HFD). The same treatment continued throughout gestation and lactation. Male offspring from each maternal group were fed the same HFD or chow after weaning and sacrificed at 13 weeks.

**Results:** Maternal SE alone increased body weight and fat mass in HFD-fed offspring, while SE+HFD offspring showed the highest energy intake and glucose metabolic disorder in adulthood. In addition, postnatal HFD increased the body weight and aggravated the metabolic disorder caused by maternal SE and SE+HFD.

**Conclusions:** Maternal HFD consumption could not ameliorate the adverse effect of maternal SE but exaggerate metabolic disorders in adult offspring. Smoking cessation and a healthy diet are needed during pregnancy to optimize the health outcome in the offspring.

## What This Paper Adds

### What Is Already Known on This Subject

Maternal smoking is one of the known risk factors for the development of obesity in offspring. A number of studies have provided evidence that maternal cigarette smoke exposure (SE) leads to low birth weight and faster weight gain during the suckling period, called catch-up growth. This fast postnatal growth is commonly observed in children, which may lead to obesity during childhood as shown in the weaning offspring in this study.

### What Important Gaps in Knowledge Exist on This Topic

Maternal high fat diet (HFD) consumption, on the other hand, can lead to *in utero* overnutrition. However, it is unclear whether in the setting of maternal SE, HFD consumption may mask the impact of *in utero* undernutrition caused by maternal SE. Furthermore, we cannot exclude the possibility that postnatal HFD consumption may worsen the regulation of energy consumption in offspring with *in utero* SE.

### What This Paper Adds

Our study demonstrated that maternal SE during pregnancy results in increased adiposity and metabolic disorders if the offspring are exposed to HFD after weaning. The additional exposure to HFD failed to counteract with cigarette SE leading to even more severe metabolic disorders in adult offspring. Therefore, both quitting smoking and maintaining a healthy diet are vital for the healthy future of the offspring.

## Key Messages

- Maternal smoking exposure (SE) decreased body weight without affecting the daily energy intake of breeders.
- Maternal HFD consumption could not ameliorate the adverse effect of maternal SE but exaggerate metabolic disorders in adult offspring.
- Postnatal HFD failed to counteract with cigarette SE but further increased the body weight and aggravated the metabolic disorder caused by maternal SE and SE+HFD.

## Introduction

Obesity is occurring at alarming rates globally, which is linked to various complications, such as metabolic disorders, diabetes, cardiovascular disease, cancers, osteoarthritis, and reproductive problems (1–3). About 2.1 billion people worldwide are estimated to be overweight or obese, increasing their risk of developing associated insulin resistance and cardiovascular disease, adding to the already enormous cost of obesity-related diseases (3, 4). The consumption of food high in energy and fat is the major driver of the obesity pandemic. However, many obese people may also have had a suboptimal intrauterine environment which may interact with obesity-induced risks.

Maternal smoking is one of the known risk factors for the development of obesity in offspring. Epidemiological investigations revealed that maternal smoking/second-hand cigarette smoke exposure (SE) during pregnancy is also a major cause of intrauterine undernutrition, even the mothers do not necessarily eat less during pregnancy compared with non-smokers (5, 6). This is caused by placental limitation, leading to pre-term birth, low body weight, and reduced head circumference at birth (7–9). However, postnatal catch-up growth results in maternal smoking being associated with an increased risk of obesity in offspring in both childhood and adulthood (10–12). Secondary to this catch-up growth, maternal SE has also been shown to contribute to later metabolic disorders in the offspring, such as glucose intolerance and type 2 diabetes, fatty liver changes, dyslipidemia, and cardiovascular disease (13–17). This has been suggested to be linked to increased eating disorders in such offspring (8, 18). Therefore, the impact of maternal SE offspring is more than just intrauterine undernutrition and fetal growth restriction, with long-lasting effects on one's adulthood.

Maternal high fat diet (HFD) consumption, on the other hand, can lead to *in utero* overnutrition. However, it is unclear whether in the setting of maternal SE, HFD consumption may mask the impact of *in utero* undernutrition caused by maternal SE. Furthermore, we can't exclude the possibility that maternal HFD consumption may worsen the regulation of energy consumption in offspring with *in utero* SE, as both can encourage energy overconsumption in rodent models (19, 20). Maternal nicotine exposure is a strong risk factor for obesogenic overeating in childhood (21). Interestingly, prenatal growth retardation during infancy has been reported in obese mothers who smoked during pregnancy (5). This indicates that HFD consumption might not be able to reverse the intrauterine undernutrition resulted from maternal smoking. In animal studies, maternal HFD consumption during pregnancy also results in increased milk intake during the suckling period, and overfeeding during this period can have additive effects to further induce fat over accumulation resulting in metabolic disorder (22, 23). However, this conclusion is difficult to apply to humans due to the complexity of dietary and smoking behaviors between different individuals, as well as other external factors.

One of the widely studied appetite regulatory networks is in the hypothalamus, consisting of neurons expressing the appetite stimulator neuropeptide Y (NPY) and its counterpart, appetite suppressors α-melanocyte stimulating hormone (α-MSH) coded by proopiomelanocortin (POMC) (24). While maternal SE may induce smoking quitting type of rebound response of NPY in the offspring's brain (6), maternal obesity leads to a heightened response of NPY signaling after fasting (25). However, it is unclear how maternal SE and maternal HFD consumption interact to influence brain appetite regulators in the offspring. Skeletal muscle is a key metabolic organ for glucose metabolism. Both maternal HFD and SE exposure during pregnancy may affect muscle genesis, and thereafter metabolic function (26, 27). Myoblast determination protein 1 (Myod1) promotes transcriptional activation of Myogenin (Myog) during myogenesis (28). It is unknown how they are affected by prenatal and postnatal insults, which were examined in this study.

Furthermore, epidemiological studies have found that HFD consumed early in life is a risk factor for childhood weight gain and later adulthood obesity, accompanied by various metabolic dysfunction (29, 30). Thus, we hypothesized that post-weaning HFD exposure may further exaggerate metabolic disorders caused by maternal SE, whereas, additional HFD exposure in the SE mothers may ameliorate the adverse impact on the metabolic regulators in the offspring. In this study, we exposed the dams to cigarette smoke with/without access to a HFD and also offered the same HFD to half of the litter after weaning. We aimed to examine the interaction between maternal and postnatal environmental factors on the metabolic outcomes in the offspring, including body weight, organ weight, gene expression of metabolic markers in the hypothalamus and metabolic organs.

## Materials and Methods

### Animals

According to the previous findings on the strain dependence of the response to SE (31), Balb/c mice were used for this study. Female mice breeders (aged 6 weeks) were housed at 20 ± 2°C in sterile micro-isolator cages and maintained on a 12:12 h light/dark cycle (lights on at 06:00 h). They were allowed a week to adapt to their new environment, with *ad libitum* access to standard rodent chow and water. The study was approved by the Animal Ethics Committee of the San Yet-sun University (number: SYSU-IACUC-2020-B0552).

### Modeling of Maternal SE and HFD Feeding

After acclimatization, female breeders were randomly divided into three groups with similar average body weight: sham exposed fed a chow (CHOW+SHAM, representing a healthy control), chow-fed SE (CHOW+SE, representing smokers consuming a balanced diet), and HFD-fed SE (HFD+SE, representing smokers consuming a “junk” diet). For SE, animals were placed inside a perspex chamber (18 L) and exposed to the smoke produced by two cigarettes (Double Happiness; Tar: 8 mg; nicotine: 0.7 mg; CO: 10 mg), twice daily for 5 weeks before mating, during pregnancy and lactation as described in Chan et al. (32). The sham exposed animals were handled identically but were not exposed to cigarette smoke. Mice were fed either standard rodent chow (3.76 kcal/g, 16% energy as fat, 20% as protein, Research Diets, Inc., United States), or a pellet HFD (4.7 kcal/g, 43% energy as fat, 20% as protein, 35% as carbohydrate, Research Diets, Inc., United States. Supplementary Table 1) (33, 34). Body weight and 24-h caloric intake were measured once a week as previously described (14, 35). After 5 weeks of pre-conditioning, females were housed and mated with male mice. The same treatment was continued until pups weaned.

### Postnatal Litter Size Adjustment and Post-Weaning HFD Feeding

On day 1 after birth, litters were adjusted to a size of 4–6 animals per litter (sex ratio 1:1) to minimize the impact of milk competition. At the age of 20 days (weaning age), male pups were used in the current study. Half of the male pups from the same litter were given a chow diet, while the other half were given the pellet HFD used in the dams. This further yielded six experimental groups, described as the maternal diet + maternal exposure – offspring diet. The six groups were CHOW+SHAM-CHOW, CHOW+SE-CHOW, HFD+SE-CHOW, CHOW+SHAM-HFD, CHOW+SE-HFD, and HFD+SE-HFD. Body weight and food intake were measured once a week until mice reached 13 weeks of age.

### Offspring IP Glucose Tolerance Test (IPGTT)

Intraperitoneal glucose tolerance test (IPGTT) was carried out as previously described (13). Briefly, 11 weeks old male offspring were fasted for 5 h. Blood samples were collected from the tail tip to establish a baseline glucose level (T_0_), which was measured again at 15, 30, 60, and 90 min after glucose injection (2 g/kg, ip). The area under the curve (AUC) was calculated for each mouse during IPGTT.

### Sample Collection

Dams were culled 1 day after pups were weaned and male offspring were culled at 13 weeks. After overnight fasting and deep anesthesia (ketamine/xylazine 180/32 mg/kg), blood was collected by cardiac puncture and blood glucose was measured immediately (Accu-Chek glucose meter, Roche) in the dams. Plasma was stored at −20°C for insulin and triglyceride (TG) measurements. Then animals were killed by decapitation and the hypothalamus was micro-dissected. Brown adipose tissue (BAT), epididymal fat, retroperitoneal (Rp) fat, mesenteric fat was dissected and weighed, as well as organs (liver and heart) and skeletal muscle [soleus, extensor digitorum longus (EDL), and tibialis]. Rp fat, BAT, and skeletal muscle were kept providing further markers of substrate metabolism.

### Plasma TG and Insulin Assays

Plasma TG was measured using glycerol standard (equivalent to 0–8.46 mM; G7793, Sigma-Aldrich) and TG reagent (T2449, Sigma-Aldrich) as previously described (35). Briefly, samples and standards were incubated with triglyceride reagent at 37°C for 20 min and read on a microplate reader (51119000, Thermo Scientific) at 490 nm. Plasma insulin concentrations were measured using a commercially available ELISA kit (KA3812, Abnova).

### Quantitative Real-Time PCR

Total RNA was isolated using TriZol reagent (15596026, Invitrogen) according to the manufacturer's instructions. The purified total RNA was used as a template to generate first-strand cDNA synthesis kit (RR036A, Takara). TaqMan probe/primers that were pre-optimized and validated by the manufacturer (only probe sequence provided by Thermo Fisher Scientific, USA, Supplementary Table 2) were used for quantitative real-time PCR (StepOnePlus Real-Time PCR System, Applied Biosystem). Markers of appetite regulation, including Npy, Npy Y1 receptor (Npy1r), Pomc, and single minded gene (Sim1), were measured in the hypothalamus. Marker involved in substrate metabolism carnitine palmitoyl-transferase (Cpt1α) and Tnfα were measured in the Rp fat. Thermogenesis markers Uncoupling protein (Ucp1 and Ucp3) were measured in BAT. Expression of muscle metabolic markers PPARγ coactivator (Pgc1α and Pgc1β), Myog and MyoD were measured in the soleus muscle.

### Statistical Methods

Results are expressed as mean ± SEM. Data on blood glucose level change during IPGTT was analyzed using one-way analysis of variance (ANOVA) with repeated measures, followed by *post-hoc* Fisher's Least Significance Difference (LSD) tests. Differences in other parameters in the dams and offspring were analyzed using one-way ANOVA and two-way ANOVA, respectively, followed by *post-hoc* LSD tests if the data were normally distributed. If not, data were log transformed to achieve normality of distribution before they were analyzed.

## Results

### SE Decreased Body Weight Without Affecting the Daily Energy Intake of Breeders

Before the start of the experiment, the average body weight of female mice was similar among the three groups. After 5 weeks of treatments, mice exposed to cigarette smoke showed significantly lower body weight than those with sham exposure (*p* < 0.05, Table 1), whereas HFD feeding increased the body weight of the mice with SE (*p* < 0.05, Table 1). This effect on body weight persisted until these breeders were sacrificed. Interestingly, SE did not affect the daily caloric intake, however, mice consumed 45.6% more calories if they were fed a HFD while exposed to cigarette smoke.

**Table 1:** Parameters of the dams.

	**Chow+Sham**	**Chow+SE**	**HFD+SE**
	**(*n* = 11)**	**(*n* = 11)**	**(*n* = 10)**
BW initial (g)	18.47 ± 0.35	18.30 ± 0.40	18.01 ± 0.39
BW at mating (g)	20.47 ± 0.11	19.07 ± 0.18^#^	22.17 ± 0.58^*^
BW at weaning (g)	24.85 ± 0.98	20.59 ±0.24^#^	24.93 ± 0.98^*^
Energy intake (kJ/d)	36.82 ± 2.16	35.95 ± 1.96	41.97 ± 5.13
Liver (mg)	1,477.8 ± 33.9	1,168.1 ± 43.7^#^	1,289.5 ± 86.1^*^
Heart (mg)	119.6 ± 3.7	111.9 ± 2.4^#^	138.0 ± 7.0^*^
BAT (mg)	51.29 ± 4.2	48.58 ± 4.61^#^	79.81 ± 5.05^*^
Rp fat (mg)	32.11 ± 13.95	16.90 ± 8.46^#^	211.9 ± 32.9^*^
Epididymal fat (mg)	240.0 ± 87.3	122.6 ± 28.0^#^	811.8 ± 195.4^*^
Mesenteric fat (mg)	461.9 ± 50.4	379.9 ± 37.5^#^	628.8 ± 78.1^*^
Glucose (mM)	9.77 ± 0.11	9.43 ± 0.16^#^	9.13 ± 1.67

*Results are showed as mean ± SEM. Data were analyzed by one-way ANOVA, followed by post-hoc LSD tests*.

# *p <0.05, maternal SE effect*;

* *p <0.05, maternal HFD effect. BAT, brown adipose tissue; BW, body weight; Rp, retroperitoneal*.

SE reduced liver and fat mass, with reduced blood glucose level (*p* < 0.05 vs. CHOW+SHAM, Table 1) which was consistent with the literature. HFD+SE dams also had increased liver and heart weight, as well as body fat such as BAT, Rp fat, epididymal fat, and mesenteric fat, while SE markedly reduced the weights of liver, heart, and fat tissues (*p* < 0.05 vs. CHOW+SE), with some even greater than the control mice, however, the glucose level was further reduced.

### Postnatal HFD Increased the Body Weight and Aggravated the Metabolic Disorder Caused by Maternal SE and SE+HFD

At weaning (postnatal day 20), CHOW+SE and HFD+SE pups appeared to have bigger body weights as compared with those from control dams (CHOW+SHAM), indicating that both maternal HFD and SE might raise the risk of obesity in young mice (Table 2).

**Table 2:** Parameters of the male offspring.

**Maternal treatments**	**CHOW+SHAM**	**CHOW+SE**	**HFD+SE**	**CHOW+SHAM**	**CHOW+SE**	**HFD+SE**
**Offspring diet**	**CHOW**			**HFD**		
	**(*****n*** **=** **16)**	**(*****n*** **=** **8)**	**(*****n*** **=** **12)**	**(*****n*** **=** **16)**	**(*****n*** **=** **8)**	**(*****n*** **=** **13)**
BW at 20 d (g)	7.01 ± 0.21	8.67 ± 0.33^#^	8.63 ± 0.30^$^	7.14 ± 0.24^*^	9.28 ± 0.31^*^^#^	8.99 ± 0.34^*^^$^
BW at 13 weeks (g)	19.71 ± 0.32	21.4 ± 0.27	20.59 ± 0.47	25.22 ± 0.66^*^	27.6 ± 0.64^*^^#^	26.99 ± 0.96^*^
Energy intake (kJ/d)	46.19 ± 8.69	46.55 ± 6.99	36.87 ± 4.81	58.51 ± 3.71	52.74 ± 2.82	64.54 ± 1.81^*^
Liver (mg)	879.9 ± 22.4	969.6 ± 15.2^#^	883.7 ± 18.5	1,055.1 ± 25.5^*^	1,093.1 ± 16.5^*^	1,149.8 ± 50.3^*^
Heart (mg)	101.3 ± 2.3	110.6 ± 3.3^#^	107.6 ± 3.6	125.0 ± 2.1^*^	137.8 ± 2.9^*^^#^	131.5 ± 2.49^*^
BAT (mg)	66.13 ± 1.26	68.4 ± 2.35	70.8 ± 2.45	118.9 ± 5.58^*^	124.0 ± 8.67^*^	134.6 ± 8.05^*^
Rp fat (mg)	125.4 ± 9.2	145.7 ± 13.8	170.3 ± 7.8	412.6 ± 38.1^*^	474.3 ± 51.4^*^	452.4 ± 39.8^*^
Epididymal fat (mg)	295.7 ± 20.0	366.0 ± 20.0	391.0 ± 19.9	906.8 ± 88.9^*^	1,101.5 ± 103.8^*^	1,189.8 ± 116.8^*^
Mesenteric fat (mg)	398.0 ± 18.2	460.8 ± 11.0	466.0 ± 27.6	592.6 ± 37.3^*^	642.2 ± 48.8^*^	704.5 ± 44.2^*^
EDL (mg)	18.70 ± 0.85	18.41 ± 0.53	17.73 ± 0.69	20.56 ± 0.46^*^	23.89 ± 0.95^*^^#^	21.29 ± 0.94^*^^$^
Soleus (mg)	10.05 ± 0.36	11.33 ± 0.56	10.81 ± 0.37	12.28 ± 0.28^*^	15.4 ± 0.79^*^^#^	12.26 ± 0.66^*^^$^
Tibialis (mg)	70.48 ± 1.58	75.58 ± 1.28	71.29 ± 2.19	80.98 ± 1.33^*^	88.66 ± 1.91^*^^#^	83.83 ± 2.41^*^

*Results are presented as mean ± SEM. Data were analyzed by multi-factor ANOVA, followed by post-hoc LSD tests*.

* *p <0.05, postnatal HFD effect*;

$ p <0.05, maternal HFD effect; and

# *p <0.05, maternal SE effect*.

*BAT, brown adipose tissue; BW, body weight; EDL, extensor digitorum longus; Rp, retroperitoneal*.

**Figure 1:**
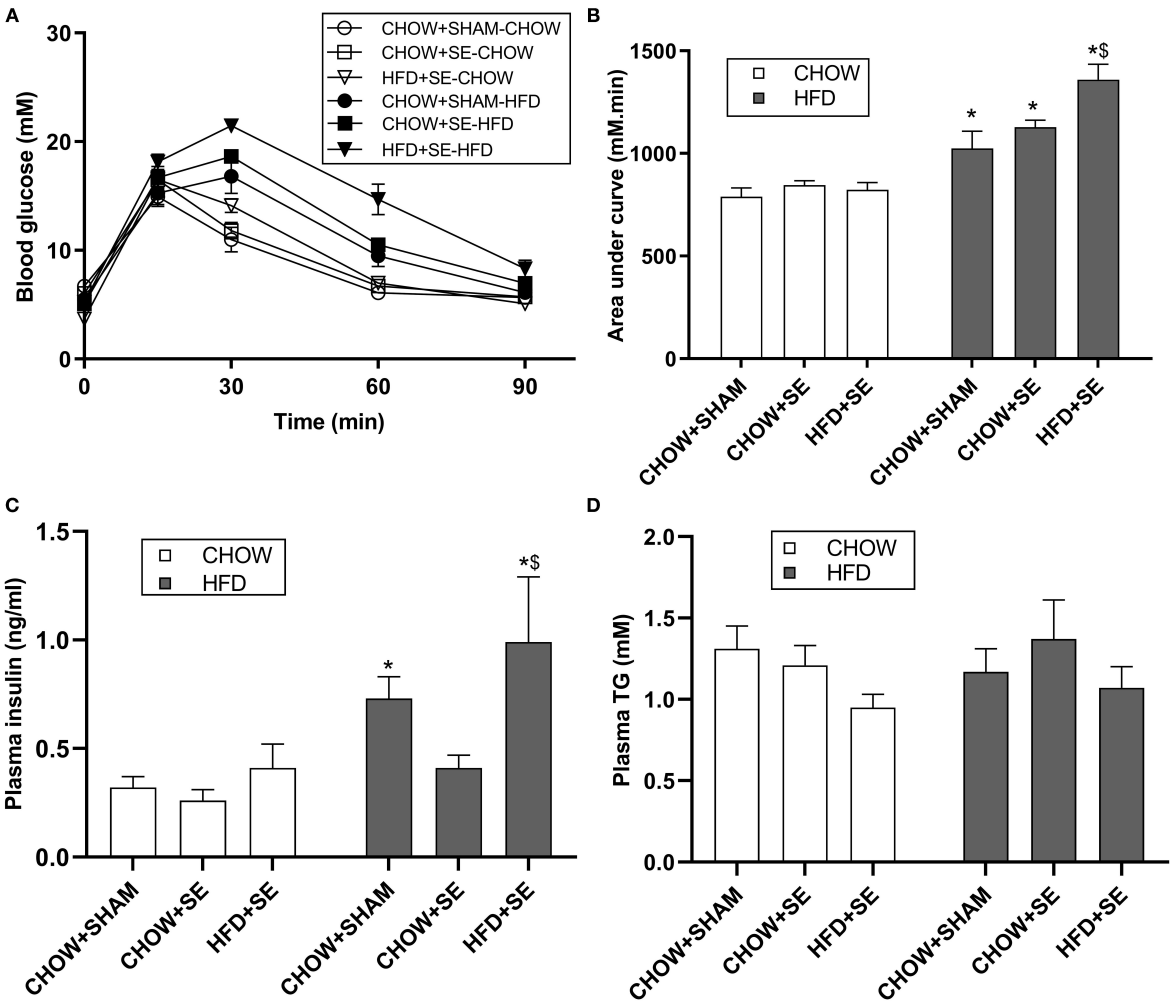
Blood glucose and lipid profile in male offspring. **(A)** Change in blood glucose levels during an IPGTT at 11 weeks. The area under the curve for panel **(A)** is shown in panel **(B)**. **(C,D)** The concentration of insulin **(C)** and TG **(D)** in plasma in male offspring at 13 weeks. All statistical results are showed as mean ± SEM. Data were analyzed by two-way ANOVA, followed by *post-hoc* LSD tests. *p < 0.05 postnatal HFD effect, ^$^*p* < 0.05 maternal HFD effects.

At 13 weeks, chow-fed offspring from CHOW+SE and HFD+SE dams showed similar body weight with only larger liver and heart in the SE-CHOW offspring. They also had a similar ability to clear blood glucose during IPGTT (Figures 1A,B), with similar plasma insulin and TG levels among the 3 chow-fed offspring groups (Figures 1C,D).

With postnatal exposure to a HFD, the body weight, body fat (BAT, RP, Epididymal, and Mesenteric fat) and skeletal muscle mass (EDL, soleus, and tibialis), were significantly greater in the offspring treatment. The mice from SE and SE+HFD dams showed a faster growth rate as compared with those from CHOW+SHAM dams (*p* < 0.05, CHOW+SHAM-HFD vs. CHOW+SE-HFD, CHOW+SE-HFD vs. HFD+SE-HFD, Table 2). A significant increase in energy intake was only observed in the HFD+SE-HFD offspring (Table 2). Moreover, CHOW+SE-HFD offspring showed significantly bigger heart and muscle weights, while HFD+SE-HFD offspring only showed larger muscle weight (EDL and Soleus) (*p* < 0.05, CHOW+SHAM-HFD vs. CHOW+SE-HFD, CHOW+SE-HFD vs. HFD+SE-HFD, Table 2). HFD+SE-HFD offspring developed more severe glucose intolerance during IPGTT than CHOW+SHAM-HFD and CHOW+SE-HFD offspring (*p* < 0.05, Figure 1B); however, only CHOW+SHAM-HFD and HFD+SE-HFD offspring showed increased plasma insulin levels (Figure 1C). In addition, plasma TG levels were not affected by postnatal HFD-consumption (Figure 1D).

Thus, maternal intervention, including SE and SE+HFD, resulted in increased body weight at weaning; however, this difference due to maternal programming diminished after consuming a balanced chow diet. While maternal SE increased body weight and heart weight without affecting adiposity when the offspring consumed a HFD, maternal exposure to both SE and HFD significantly increased caloric intake and resulted in the largest fat mass although without statistical significance.

### Effects on Hypothalamic Appetite Regulators in Male Offspring

**Figure 2:**
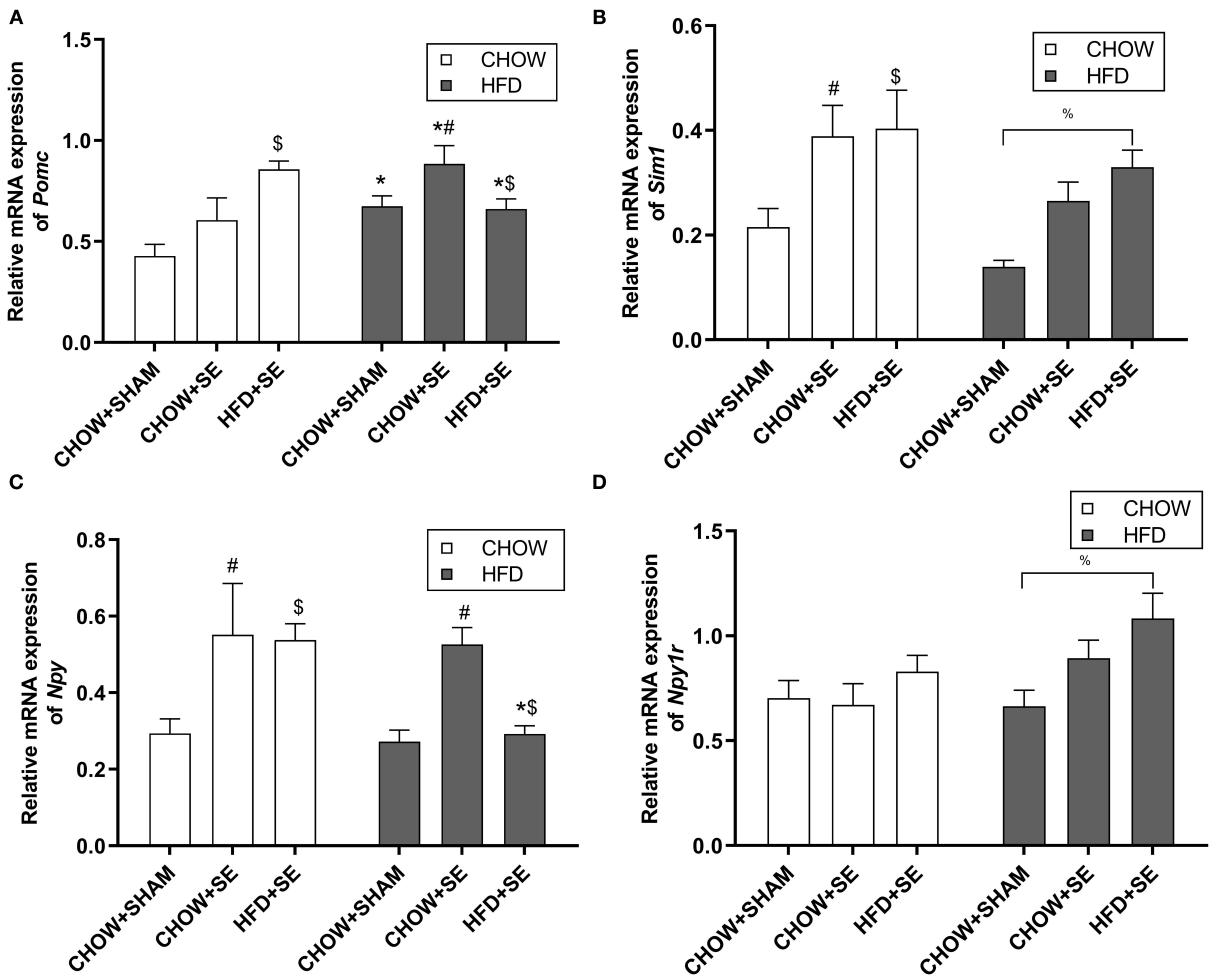
Expression of the energy homeostatic regulator in the hypothalamus. Hypothalamic mRNA expression of *Pomc* **(A)**, *Sim1* **(B)**, *Npy* **(C)**, and *Npy1r* **(D)** in the male offspring at 13 weeks. Results are expressed as mean ± SEM. Data were analyzed by two-way ANOVA, followed by *post-hoc* LSD tests. *p < 0.05, postnatal HFD effect; ^$^*p* < 0.05, maternal HFD effect; and ^#^*p* < 0.05, maternal SE effect. ^%^*p* < 0.05.

To investigate the effects of the maternal and postnatal HFD consumption on the feeding regulators, we checked the mRNA expression in the hypothalamus. In chow-fed offspring, *Pomc* mRNA expression was significantly up-regulated in the HFD+SE-CHOW group (*p* < 0.05 vs. CHOW+SHAM, Figure 2A), whereas *Sim 1* was significantly increased in the CHOW+SE-CHOW and HFD+SE-CHOW groups (*p* < 0.05 vs. CHOW+SHAM, Figure 2B). Moreover, hypothalamic *Npy* mRNA was only increased in CHOW+SE-CHOW offspring (*p* < 0.05 vs. CHOW+SHAM, Figure 2C), although the level in the HFD+SE-CHOW group was comparable to the CHOW+SE-CHOW group. However, *Npyr1r* mRNA was similar among chow-fed offspring.

In HFD-fed offspring, *Pomc* mRNA expression was increased by HFD consumption (*p* < 0.05 vs. CHOW+SHAM-CHOW, Figure 2A), which was further increased in CHOW+SE-HFD mice (*p* < 0.05, CHOW+SE-HFD vs. CHOW+SHAM-HFD). There was no difference in *Pomc* mRNA level between HFD+SE-HFD and CHOW+SE-HFD group. However, *Sim 1* was only significantly increased in the HFD+SE-HFD group compared with the CHOW+SHAM-HFD group (*p* < 0.05, Figure 2B). *Npy* was significantly upregulated in the CHOW+SE-HFD group (*p* < 0.05 vs. CHOW+SHAM-HFD, Figure 2C) although similar to its chow-fed littermates, whereas *Npyr1r* mRNA was significantly upregulated in the HFD+SE-HFD offspring (*p* < 0.05 vs. CHOW+SHAM-HFD, Figure 2D).

### Effects on the Substrate Metabolic in the Fat and Muscle in the Offspring

**Figure 3:**
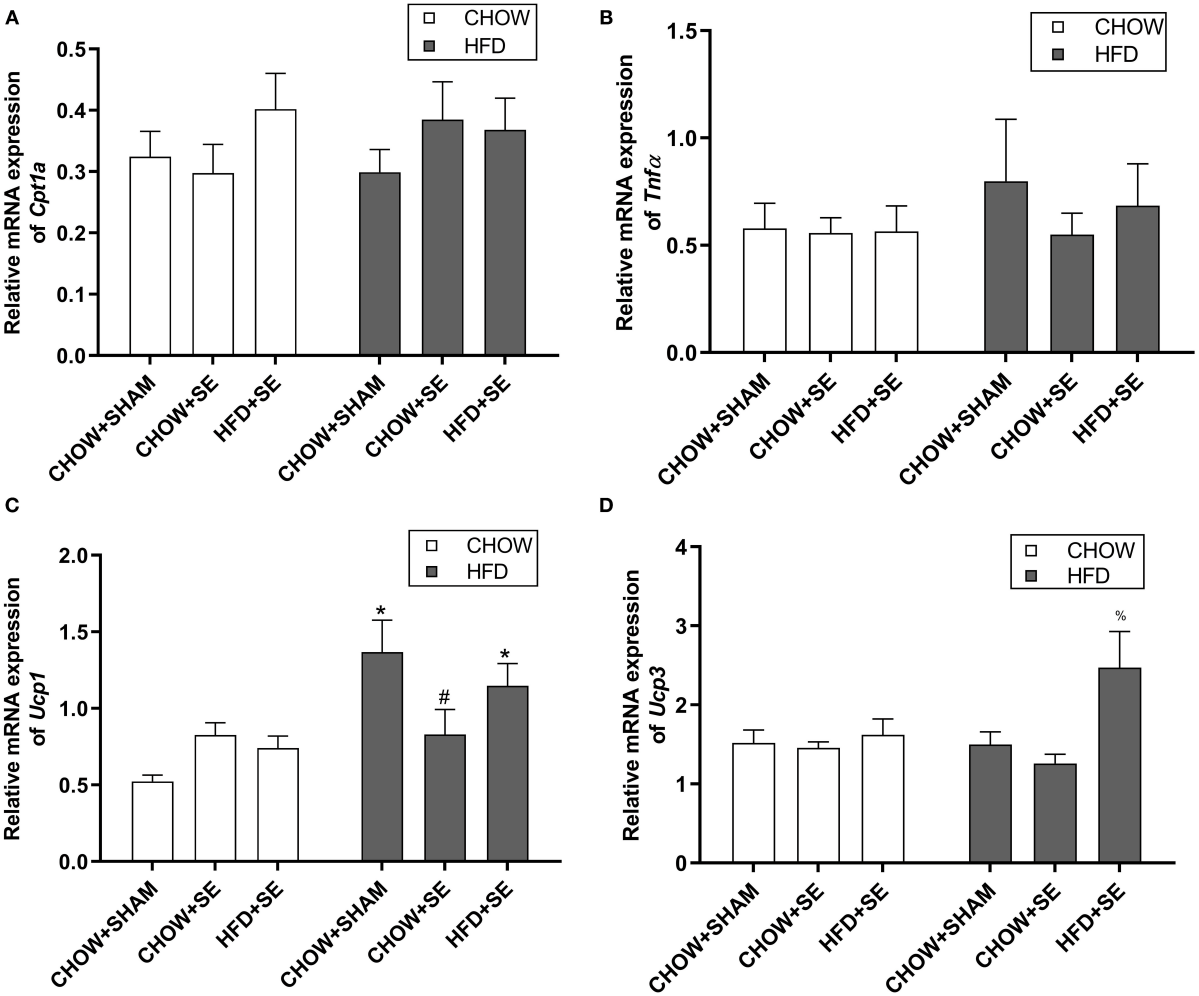
Expression of energy metabolic regulators in fat tissue. mRNA expression of *Cpt1*α **(A)**, *Tnf*α **(B)** in the white fat and, *Ucp1* **(C)**, and *Ucp3* **(D)** in the brown fat in the male offspring 13 weeks. Results are expressed as mean ± SEM. Data were analyzed by two-way ANOVA, followed by *post-hoc* LSD tests. *p < 0.05, postnatal HFD effect and ^#^*p* < 0.05, maternal SE effect. ^%^*p* < 0.05 vs. CHOW+SHAM-HFD and CHOW+SE-HFD.

Cpt1α is the rate-limiting step for fatty acid oxidation in the mitochondrial, while Tnfα is a pro-inflammatory cytokine which plays a key role in insulin resistance. The expression of *Cpt1*α and *Tnf*α were no affected by neither maternal nor postnatal interventions (Figures 3A,B). However, the expression of the thermogenesis markers *Ucp1* was significantly up-regulated by postnatal HFD consumption in CHOW+SHAM-HFD and HFD+SE-HFD groups compared with their chow-fed littermates (*p* < 0.05, Figure 3C), while *Ucp3* was significantly higher in the HFD+SE-HFD compared with CHOW+SHAM-HFD and CHOW+SE-HFD groups (*p* < 0.05, Figure 3D).

**Figure 4:**
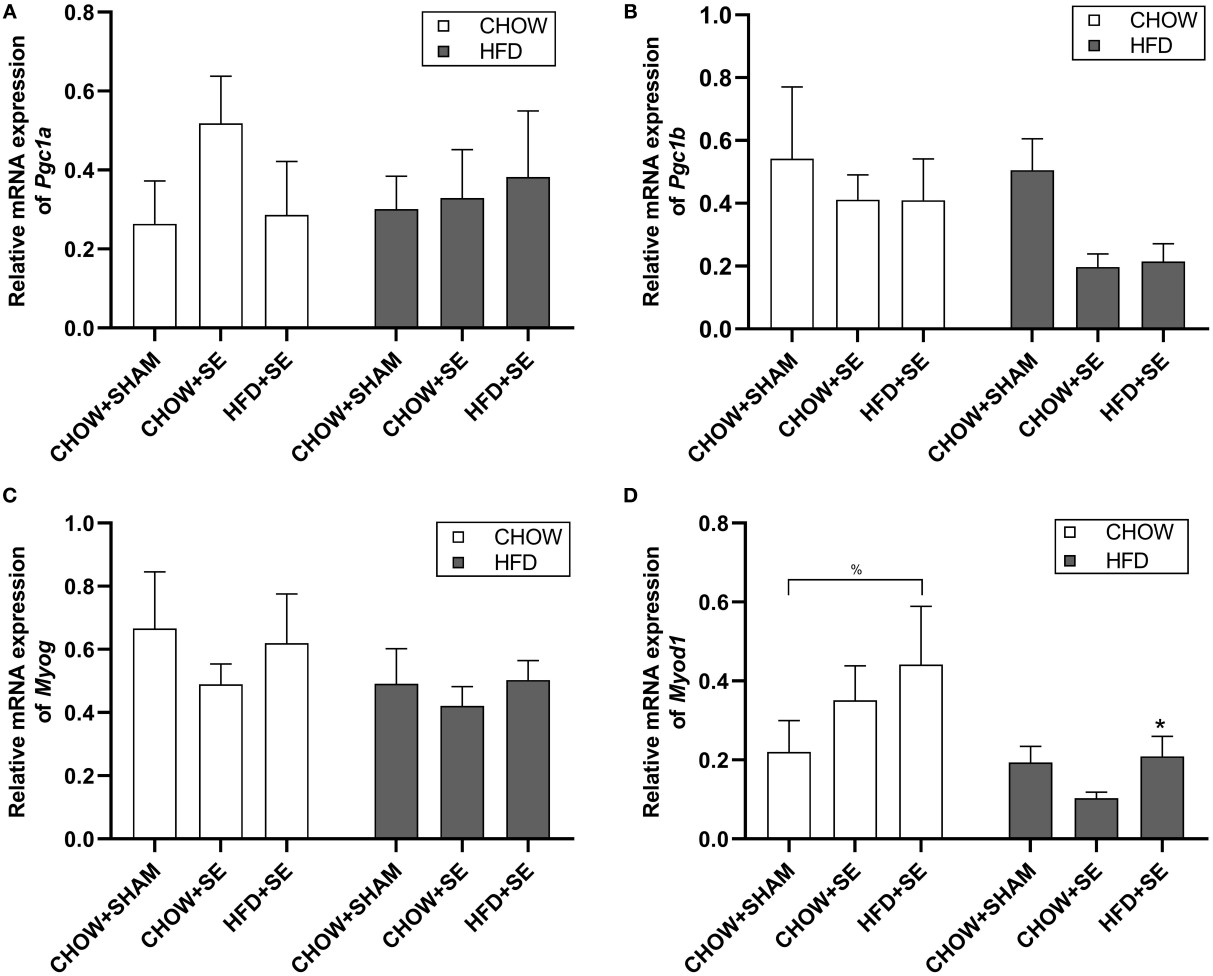
Expression of the metabolic regulator and myogenesis related gene in skeletal muscle tissue. Muscle mRNA expression of *Pgc1*α **(A)**, *Pgc1*β **(B)**, *Myog* **(C)**, and *Myod1* **(D)** in the male offspring at 13 weeks. Results are expressed as mean ± SEM. Data were analyzed by two-way ANOVA, followed by *post-hoc* LSD tests. *p < 0.05, postnatal HFD effect; ^%^*p* < 0.05.

In soleus muscle, we check the expression of mitochondrial biogenesis markers Pgc1α and Pgc1β, which were not significantly affected by maternal programming nor postnatal HFD consumption (Figures 4A,B), albeit a trend decrease in *Pgc1*β expression in CHOW+SE-HFD and HFD+SE-HFD groups. Furthermore, *Myog* expression was not different among all groups (Figure 4C); however, *Myod1* expression was up-regulated in the HFD+SE-CHOW group (*p* < 0.05 vs. CHOW+SHAM-CHOW), which was further reduced by postnatal HFD feeding (*p* < 0.05 vs. HFD+SE-CHOW, Figure 4D).

## Discussion

In this study, we demonstrated that maternal SE exaggerates metabolic disorders if the offspring consume a HFD after weaning. The additional HFD consumption did not ameliorate or reverse the adverse effects due to maternal SE. On the contrary, maternal exposure to both SE and HFD led to the worst outcome in the offspring fed a HFD after weaning.

Exposure to cigarette smoke alone induced significant wasting in the dams, reflected by reduced both fat and lean body mass, consistent with a previous study (13). While additional HFD consumption only significantly increased the body weight at mating, after lactation, the body weight of HFD+SE dams was comparable to the control offspring. However, abdominal fat masses were increased in mice exposed to cigarette smoke at this time point. This is consistent with observations in humans that smoking encourages the development of central obesity albeit smaller body weight (36).

A number of studies have provided evidence that maternal SE led to low birth weight and faster weight gain during the suckling period, called catch-up growth (6, 37, 38). This fast postnatal growth is commonly observed in children, which may lead to obesity during childhood as shown in the weaning offspring in this study (39, 40). Childhood BMI can positively correlate with that in adulthood, which has been well-represented in the offspring with *in utero* SE. This is not caused by overeating, as the caloric intake was similar between CHOW+SE-HFD and CHOW+SHAM-HFD offspring. Maternal nicotine exposure has been shown to reduce hypothalamic *Npy* and increase *Pomc* expression in newborns (6). The increase in both in the CHOW+SE-HFD offspring may reflect a withdrawn rebound, similar to the response in the smokers after quitting. This may be attributed to larger fat mass in CHOW+SE-HFD offspring. There are several homologs of UCPs, including UCP1 and UCP3, which are responsible for mediating thermogenesis and basal metabolic rate in fat (41). Previously study showed that caloric restriction could down-regulate UCP1 to and UCP3 reserve energy expenditure (42, 43). Thus, the HFD leads to an adaptive upregulation of UCPs to increase energy expenditure as shown in CHOW+SHAM-HFD mice. Compared with CHOW+SHAM-HFD offspring, those with intrauterine SE had suppressed thermogenesis marker UCP1 in their BAT, which may impair the adaptive increase in heat production observed in CHOW+SHAM-HFD offspring, resulting in increased fat mass in CHOW+SE-HFD offspring. Glucose intolerance in CHOW+SE-HFD mice may be due to insulin insufficiency, rather than insulin resistance in CHOW+SHAM-HFD, as reflected by plasma insulin levels in these two groups. Increased insulin level is a sign of insulin resistance. HFD has been shown to reduce insulin sensitivity and promote the development of type 2 diabetes (44). Previous studies have shown that maternal nicotine treatment can interrupt β-cell functions in the offspring (45–47).

“Eat for two” is a traditional practice for pregnant women even in the current obesity pandemic. As intrauterine undernutrition is a key to maternal smoking, such practice may be more appealing. However, in this study, we have shown that the combination of maternal HFD and SE further exaggerates the metabolic disorders than maternal SE alone when the offspring were exposed to an obesogenic environment after birth. There are several mechanisms suggested in this study. Firstly, overeating was only observed in HFD+SE-HFD offspring, which may be driven by markedly upregulated Npy1r, the orexigenic receptor for NPY. This increase in activity was not counteracted by the adaptive increase in Sim1 expression which lies downstream of the receptor of the anorexigenic peptide αMSH. Secondly, the heightened glucose intolerance may be a combination of impaired β-cell function due to maternal SE and insulin resistance due to both intrauterine and postnatal HFD exposure. Albeit overeating, the body weight and fat mass in HFD+SE-HFD seem to be controlled. This may be due to more than doubled Ucp3 expression to increase the energy expenditure while energy was over consumed.

During the weight gain by HFD consumption, muscle mass is also increased to support the increased body weight, as shown in HFD-fed mice in this study. The greatest increase in HFD+SE-HFD mice may be proportional to their body weight. Pgc1α controls mitochondrial biogenesis and angiogenesis in skeleton muscle (48–50); whereas, Pgc1β activates an anti-angiogenesis gene program in the skeletal muscle and its overexpression can induce muscle wasting by inhibiting ubiquitin-mediated proteolysis (51, 52). In our present study, the trending decrease in Pgc1β in mice from SE dams can help to explain the increased muscle mass. However, this did not seem to lead to an improvement in muscle metabolic function. Previous studies suggested that Myod1 could promote transcriptional activation to regulate the expression of muscle-specific genes, including Myog, which plays a crucial role in the terminal differentiation during myogenesis (28, 53). In this study, we found significantly altered *Myod1* expression but not *Myog* by maternal HFD+SE. This may be an adaptation to prevent a reduction in muscle mass by maternal SE, which is common in smokers (54). However, the Myod1 changes in offspring from HFD+SE dams was reversed by postnatal HFD consumption. This may be due to myogenesis, as we observed in the other groups fed a HFD. However, further studies are needed to examine the metabolic functions of the muscle as well as mitochondrial function which is beyond the scope of this study.

Whilst there was increased fat mass and unchanged fatty acid metabolic marker Cpt1α, plasma TG was not affected by maternal programming nor postnatal HFD consumption, which might be due to the mouse strain specific. It has previously been shown that with cigarette SE only during lactation, there were decreased proteins levels of Ucp1 and Cpt1 and reduced sympathetic nerve stimulation upon BAT in female adult rat (55). The same was observed with the administration of isolated nicotine through minipumps on the dams at the same period (56). Similarly, in other rat model of SE only during lactation, SE group male adult offspring did not changed insulinemia, despite higher serum glucose levels, suggesting a pancreatic insulin secretory failure (57). Nevertheless, it needs to be noted that none of the above metabolic abnormalities were observed in the chow-fed offspring. This highlights the importance of a healthy diet to prevent the adverse impact of maternal programming on metabolic outcomes in adulthood. As this study was only performed with male mice, it cannot be directly extended to the female offspring, due to the sexual differences during maternal programming as shown by the others (13, 58). In a similar model of SE, females offspring showed increased glucose tolerance by maternal SE, which is consistent with our founding (13). In addition, another study showed that female offspring from the SE dams had lower levels of anorexigenic neuropeptides, Cocaine-, and amphetamine-regulated transcript and MSH, in their brains (59).

In conclusion, this study demonstrated that maternal SE during pregnancy results in increased adiposity and worsened metabolic disorders if the offspring are exposed to HFD after weaning. The additional maternal exposure to HFD interacts with SE which exacerbated metabolic disorders in the male offspring by disrupting metabolic regulators. Therefore, both quitting smoking and maintaining a healthy diet are vital for the healthy future of the offspring.

## Data Availability Statement

The raw data supporting the conclusions of this article will be made available by the authors, without undue reservation.

## Ethics Statement

The animal study was reviewed and approved by Institutional of Animal Care and Committee of San Yet-sun University.

## Author Contributions

TH: conceptualization, methodology, visualization, investigation, and writing – original draft. MY and YZ: investigation and editing. XH: investigation. NW: visualization and investigation. YC, PL, and JY: supervision and validation. CC and BO: supervision and review. CY: supervision, writing, review, and editing. All authors contributed to the article and approved the submitted version.

## Conflict of Interest

The authors declare that the research was conducted in the absence of any commercial or financial relationships that could be construed as a potential conflict of interest.
