# Supplementary material for: Maternal High Fat Diet Consumption Exaggerates Metabolic Disorders in Mice With Cigarette-Smoking Induced Intrauterine Undernutrition
Source: Front Nutr. 2021 Mar 16;8:638576. doi: 10.3389/fnut.2021.638576 (PMC8007928; doi:10.3389/fnut.2021.638576)
Supplement: Supplementary file 1 [file Data_Sheet_1.docx]

**Maternal high fat diet consumption exaggerates metabolic disorders in mice with intrauterine undernutrition due to cigarette smoke exposure**

*Taida Huang^1,2*^, Mo Yang^1*^, Yunxin Zeng^1^, Xiaomin Huang^1^,Nan Wang^1^, Yun Chen^1^, Peng Li^1^, Jinqiu Yuan^1^, Chun Chen^1&^, Brian G Oliver^3,4#^, Chenju Yi^1&#^*

1. Research Center, The Seventh Affiliated Hospital of Sun Yat-sen University, Shenzhen, 518107, China;

2. School of Biomedical Sciences, Faculty of Medicine, The Chinese University of Hong Kong, Hong Kong SAR, China;

3. School of Life Sciences, Faculty of Science, University of Technology Sydney, NSW 2007, Australia;

4. Respiratory Cellular and Molecular Biology, Woolcock Institute of Medical Research, NSW 2037, Australia

***contribute equally**

**# joint-senior author**

**& Corresponding authors**

**Supplementary Table 1. Nutritional composition of the high fat diet (Research Diets, Inc., United Status)**

| Casein, Lactic, 30 Mesh | 200.00 g |
| --- | --- |
| Cystine, L | 3.00 g |
| Sucrose, Fine Granulated | 176.80 g |
| Lodex 10 | 100.00 g |
| Starch, Corn | 72.80 g |
| Solka Floc, FCC200 | 50.00 g |
| Lard | 177.50 g |
| Soybean Oil, USP | 25.00 g |
| S10026B | 50.00 g |
| Choline Bitartrate | 2.00 g |
| V10001C | 1.00 g |
| Dye, Red FD&C #40, Alum. Lake 35-42% | 0.05 g |
| Total: | 858.15 g |

**Supplementary Table 2. Probe sequences (Thermo Fisher Scientific) used in real-time PCR.**

| **Gene** | **Assay ID** | **Probe sequence (5’→3’)** |
| --- | --- | --- |
| *Pomc* | Mm00435874_m1 | AGCAACCTGCTGGCTTGCATCCGGG |
| *Sim1* | Mm00441390_m1 | TCTTTCCAGAAGGGCTTGGCGAGGC |
| *Npy* | Mm00445771_m1 | TCATCACCAGACAGAGATATGGCAA |
| *Npy1r* | Mm00650798_g1 | TTTATATTCATATGCTACTTCAAGA |
| *Cpt1α* | Mm00550438_m1 | TACCGTGAGCAGGTACCTGGAGTCT |
| *Tnfα* | Mm00443259_g1 | CCCTCACACTCAGATCATCTTCTCA |
| *Ucp1* | Mm00494069_m1 | CCAAAGTCCGCCTTCAGATCCAAGG |
| *Ucp3* | Mm01163394_m1 | GTGGAAAGGGACTTGGCCCAACATC |
| *Pgc1α* | Mm00447183_m1 | CGCAACATGCTCAAGCCAAACCAAC |
| *Pgc1β* | Mm00504720_m1 | TCTCTCTGACACGCAGGGTGGGGAC |
| *Myog* | Mm00446194_m1 | CGGGCCCCAGCCCATGGTGCCCAGT |
| *Myod1* | Mm00440387_m1 | TGATGGCATGATGGATTACAGCGGC |

Pomc: proopiomelanocortin; Sim1: Single-minded homolog 1; Npy: Neuropeptide Y; Npy1r: Neuropeptide Y Receptor Y1; Cpt1α: carnitine palmitoyltransferase 1A; Tnfα: Tumor Necrosis Factor-alpha; Ucp1: Uncoupling Protein-1; Ucp3: Uncoupling Protein-3; Pgc1α: PPARγ coactivator-1 alpha; Pgc1β: PPARγ coactivator-1 beta; Myog: Myogenin; Myod1: Myogenic Differentiation 1.
